# Access to End-of Life Parkinson's Disease Patients Through Patient-Centered Integrated Healthcare

**DOI:** 10.3389/fneur.2018.00627

**Published:** 2018-07-30

**Authors:** Carsten Eggers, Richard Dano, Juliane Schill, Gereon R. Fink, Lars Timmermann, Raymond Voltz, Heidrun Golla, Stefan Lorenzl

**Affiliations:** ^1^Department of Neurology, University Hospital of Cologne, Cologne, Germany; ^2^Department of Neurology, Philipps-University Marburg, Marburg, Germany; ^3^Cognitive Neurology Section, Research Center Juelich, Institute of Neuroscience and Medicine, Juelich, Germany; ^4^Department of Palliative Medicine, University Hospital of Cologne, Cologne, Germany; ^5^Center for Integrated Oncology, Cologne, Germany; ^6^Center for Clinical Studies (ZKS), University Hospital Cologne, Cologne, Germany; ^7^Endowed Professorship for Interdisciplinary Research in Palliative Care, Institute of Nursing Science and Practice, Paracelsus Medical University, Salzburg, Austria; ^8^Department of Palliative Care, Klinikum der Universität München, Ludwig-Maximilians-University, Munich, Germany

**Keywords:** Parkinson, palliative care, end-of-life, integrated care, late-stage, network

## Abstract

**Background:** Palliative care in Parkinson's Disease (PD) patients considerably differs from palliative care in oncology patients. Integrated care models are a concept to support patients and improve management of PD symptoms. However, it is not known if the access to PD patients at the end of life can be achieved through integrated care models.

**Aim:** To analyze an integrated model of care for PD patients with the aim to identify if this integrated model of care has access to PD patients at the end of life.

**Material and Methods:** The Cologne Parkinson's network was designed as a randomized, controlled prospective clinical trial in order to increase quality of life of PD patients. This innovative model of care integrated a neurologist in private practice, a movement disorder specialist of the University Hospital and a PD nurse. Mortality rates of PD patients during the study period of 6 months were registered and compared with mortality rates of the general population of Germany according to the Federal Statistical Office of Germany. The retrospective *post-hoc* analysis was conducted after completion of the initial study at the University Hospital and neurologists' practices in the greater area of Cologne, Germany. Eligible patients had a diagnosis of idiopathic PD and were aged 25–85 years.

**Results:** Parkinson's Disease patients in this trial had an even slightly lower mortality rate as the general population (1.66 v. 2.1%). These results are contradictory and speak for a substantial proportion of late-stage disease patients, who have not been adequately included in this study or have been better treated within this trial. The mean disease duration of patients in this study was around 6 years which resembles the lower range of the mean disease duration at death of PD patients in general.

**Conclusions:** The results of our *post-hoc* analysis show, that accessing PD patients in the last phase of their disease is extremely difficult and nearly fails in spite of an integrated care approach. Reasons for poor access and loss of follow-up at the end of life have to be identified and care models for PD patients until the end of life should be developed urgently.

## Introduction

Despite a significant progress in treatment strategies and modern therapy concepts neurodegenerative diseases like idiopathic Parkinson's disease (PD) or atypical Parkinsonian disorders inevitably lead to progressive motor, neuropsychiatric and non-motor symptoms (1–5). Dementias develops in up to 80% of patients after 20 years (6), depressions in more than 40% of patients and psychotic experiences are frequent in PD patients (7). Reduced mobility implicates higher mortality as in the age-related population, specifically due to infections (pneumonia, urinary tract infections) or falls with consecutive fractures (8). According to a recent meta-analysis, mortality in PD patients is increased in a range of 0.9–3.8. The mean duration until death ranges between 6.9 and 14.3 years, where increasing age and development of dementia were most commonly associated with increased mortality (9).

Palliative care in PD patients considerably differs from palliative care provision in oncology patients, in terms of the models of care, the provision and the duration. The beginning of the palliative phase in PD is still not well defined but according to a recent publication it lasts about 2.2 years for PD patients and 1.5 years for APS before death (10). Currently only occasionally palliative care structures are integrated selectively during the course of the disease. Patients with PD/APS die from infections as a consequence of swallowing difficulties or injuries and fractures as the consequences of falls (11), but hardly ever in hospices and more seldom at home than patients of other oncological diseases (12).

In the last years in the greater area of Cologne, Germany, the Cologne Parkinson's network was designed as a randomized, controlled prospective clinical trial in order to increase quality of life of PD patients. This innovative model of care integrated a neurologist in private practice, a movement disorder specialist and a PD nurse of the University Hospital. In consultation hours at the practices of the neurologists' patients met with the integrated care team and individual neurological treatment plans were designed. The PD nurse visited patients at home regularly every 3 months and could be contacted in between to follow and address patients' Parkinson-related problems. This integrated, multiprofessional, individual and personalized therapy meeting individual needs of patients improved their quality of life, motor functioning as well as non-motor symptoms (13).

This integrated care model included PD patients, who were able to visit a practice of a neurologist. Our retrospective *post-hoc* analysis of the trial's data aimed to detect whether this care model managed to access or follow, respectively, also PD patients at the end of life.

## Materials and methods

This study was set up as a randomized controlled prospective clinical study with two arms in the greater area of Cologne in Germany.

The Cologne Parkinson Network (CPN) was established together with movement disorders experts and a PD nurse from the University Hospital of Cologne, Department of Neurology (CE) together with 25 community neurologists.

The trial was conducted between February 2012 (first patient first visit) and July 2015 (last patient last visit) and was approved by the local ethics committee of the medical faculty of the University of Cologne (No. 11-233). For further details of this trial we refer to the published study (13). The study was registered in the German Register for Clinical studies (DRKS00003452).

Briefly, patients were screened for potential involvement [age 25–85 years, exclusion criteria were unstable medical condition as a co-morbidity, major depression (BDI-2 >30 points), severe cognitive decline (PANDA <14 points)] by community neurologists and presented in quarterly Parkinson's consultation hours together with the movement disorders expert and the PD nurse. The time of the consultation was set as needed (up to a maximum of 45 min). Patients were randomized to either a control group (CG) or an intervention group (IG). In the CG, patients were included in the study at the baseline visit in the Parkinson's consultation hour and continued regular German neurological treatment. This included visits at the community neurologists practice about every 3 months (baseline, 3 months, 6 months). Once included, the PD nurse obtained questionnaires and surveyed clinical parameters (e.g., UPDRS III) at baseline and every 3 months. Patients had access to regular physiotherapy, occupational or speech therapy. Access to different medications was the same for both treatment arms.

The IG-treatment additionally included the development of an individual treatment plan, regular home visits of a PD nurse (every 3 months or whenever necessary on short notice) and a telephone hotline. Individual treatment plans were reviewed every 4 weeks and adapted according to individual patients' needs. Furthermore, the PD nurse synchronized the therapeutic pharmacological intervention with the program of speech therapists or physiotherapists. Thus, whenever necessary, rapid therapeutic modifications could be achieved.

Primary outcome parameter was the PDQ-39 to assess quality of life of patients. Changes in mood, motor and non-motor functioning and cognition (BDI-2, UPDRS III, NMS-Score, PANDA) were evaluated as secondary outcome parameters. Daily medication was converted to the Levodopa equivalence dose according to published conversion rates (14).

Mortality rates of patients during the study period of 6 months were registered and compared with mortality rates of the general population of Germany according to the Federal Statistical Office of Germany (www.destatis.de).

## Results

**Figure 1:**
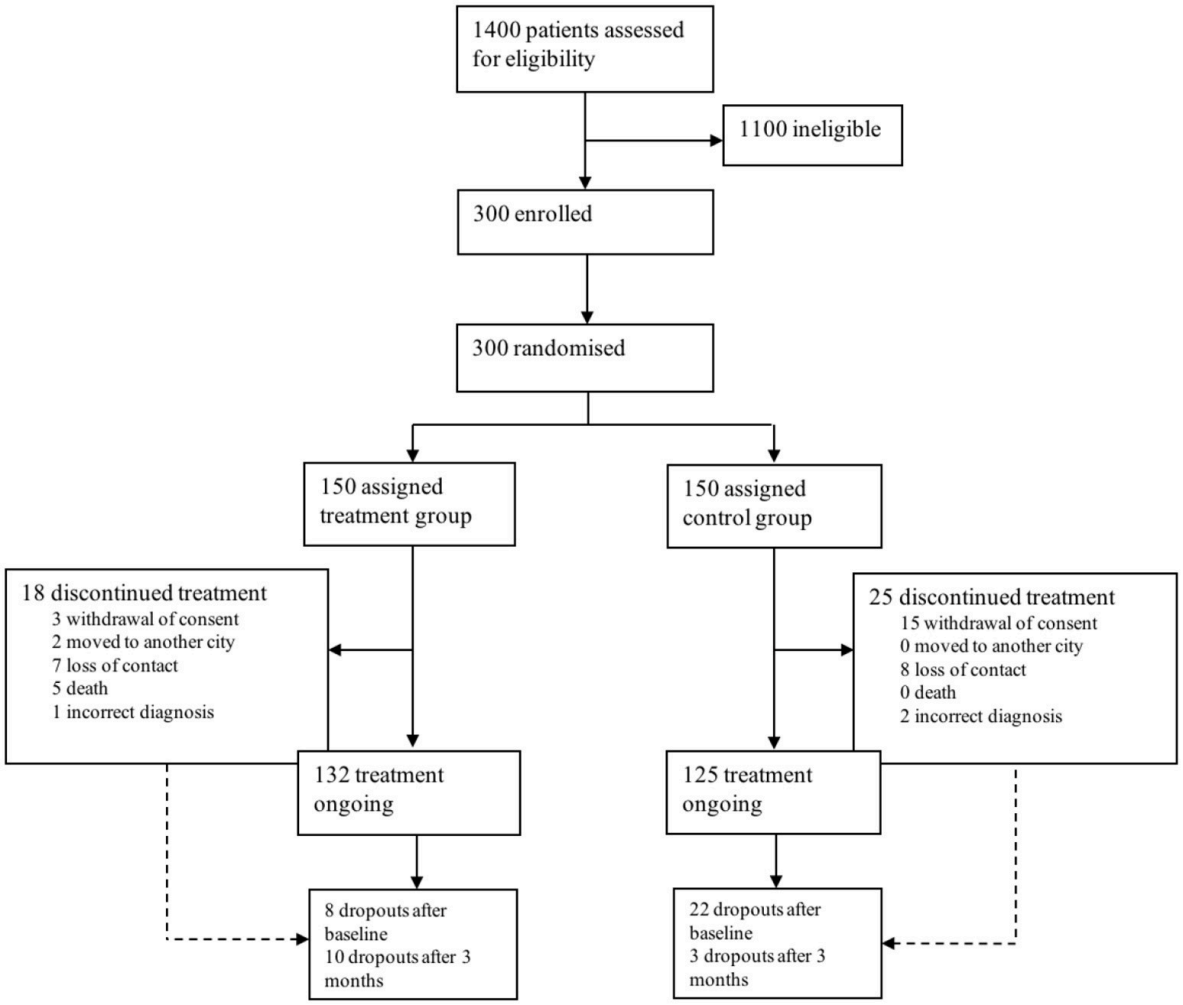
Trial profile.

A total of 1,400 patients were screened for eligibility. 300 patients were eligible, included and randomized. Patients were equally randomly assigned to an intervention (IG) and control group (CG). Mean age at baseline was 69.8 ± 8.4 for the IG and 69.9 ± 7.8 years in the CG. 132 patients in the IG and 125 in the CG completed the study, 37 patients dropped out (see Figure 1 for reasons). Overall, 5 patients deceased during the study period in the IG, which is 1.66% of the total study population (*n* = 300). Reasons for death were heart failure due to myocardial infarction (*n* = 3), hospitalization after femoral neck fracture, secondary aspiration pneumonia and sepsis (*n* = 1) and in consequence of pancreatic cancer (*n* = 1). None of the patients in the CG deceased.

PDQ-39 improved more in IG compared to CG (2.2 points (95% CI −4.4 to 0.1); *p* = 0.044). Likewise, change scores between IG and CG favored IG for UPDRS III (*p* < 0.001, mean change 3.3, 95% CI −4.9 to −1.7) and PD-NMS (*p* < 0.001, mean change 11.3, 95% CI −17.1 to −5.5).

The primary outcome parameter significantly improved in the IG compared to the CG over a 6-month period (2.2 points (95% CI −4.4 to 0.1); *p* = 0.044). The secondary outcome UPDRS improved in the IG after 6 months (*p* < 0.001, mean change 3.3, 95% CI −4.9 to −1.7). The scores of the PD-NMS improved likewise after 6 months in favor of the IG (*p* < 0.001, mean change 11.3, 95% CI −17.1 to −5.5). No changes were detected for the cognition (PANDA) or depressive symptoms (BDI-2). For an overview of baseline characteristics see Table 1.

**Table 1:** Baseline characteristics IG and CG.

**Outcome parameter**	**Intervention group (IG) Mean and SD**	**IG *n* =**	**Control group (CG) Mean and SD**	**CG *n* =**	*p*-value^*^
Age in years	69.8 ± 8.4	131	69.9 ± 7.8	132	0.924
Women/men		47/85		52/80	0.518
Disease duration/time since diagnosis in years	6.2 ± 6.2	126	5.5 ± 5.2	124	0.716
Hoehn and Yahr stage	2.5 ± 0.8	132	2.6 ± 0.8	125	0.687
**Primary Outcome: PDQ-39 Total Score**	26.0 ± 14.8	132	27.7 ± 15.6	125	0.407
Subscale mobility	32.1 ± 26.6	132	31.9 ± 24.3	125	0.882
Subscale activities of daily living	27.9 ± 23.6	132	28.9 ± 23.2	125	0.661
Subscale emotional well-being	27.3 ± 20.9	132	31.9 ± 19.6	125	0.072
Subscale stigma	17.4 ± 16.1	132	19.6 ± 20.2	125	0.815
Subscale social support	14.2 ± 19.6	132	14.5 ± 18.7	125	0.561
Subscale cognition	30.9 ± 19.6	132	33.3 ± 21.1	125	0.436
Subscale communication	22.0 ± 18.8	132	2.9 ± 21.1	125	0.927
Subscale bodily discomfort	36.0 ± 23.0	132	39.2 ± 23.4	125	0.266
**SECONDARY OUTCOMES**					
UPDRS III	28.3 ± 9.1	132	28.0 ± 8.7	125	0.938
PANDA	24.7 ± 3.8	131	24.7 ± 3.5	125	0.795
BDI-2	12.0 ± 8.2	132	12.6 ± 7.3	125	0.266
NMS	53.9 ± 29.6	132	62.3 ± 34.6	125	0.057
Daily LEDD	612.9 ± 431.3	132	612.4 ± 390.6	125	0.659
**OTHER**					
**Medication use in %**					
Levodopa	34%	132	34%	125	0.921
Dopamine agonist	30%	132	31%	125	0.885
COMT inhibitor	9%	132	9%	125	0.927
MAO B blocker	16%	132	15%	125	0.862
Amantadin	11%	132	11%	125	0.911
Anticholinergic	0%	132	0.71%	125	0.101
Deep brain stimulation	4%	132	2.8%	125	0.422

* *The p-values are from Pearson's chi-square test (nominal data) or Kruskal-Wallis test (at least ordinal data), respectively*.

According to the mortality tables of the general population the mean mortality rate for the years 2012–2014 is 2.1% (mean of yearly mortality rates for women/men) for citizen aged 60–80 years (as comparable to the set of the study patients: mean age of patients ± standard deviation) https://www.destatis.de/DE/ZahlenFakten/GesellschaftStaat/Bevoelkerung/Sterbefaelle/Sterbefaelle.html;jsessionid=CC24B4774EDE040EE924FA2B881F0EE9.cae4%22%20/l%20%22Tabellen%22). As such, the group of PD patients in this trial had an even lower mortality rate as the general population (1.66 v. 2.1%).

## Discussion

This integrated care model was implemented including various modalities to sustain quality of life in PD patients. The primary and secondary outcome parameters were adequately achieved in this study. Furthermore, this approach may have the opportunity to improve access to PD patients also at the end of life. However, the results of our *post-hoc* analysis show, that accessing PD patients in the last phase of their disease is extremely difficult and nearly fails in spite of an integrated care approach. Reasons for loss of follow-up have to be identified and care models for PD patients until the end of life should be developed urgently. In this study, one major reason for poor access to and loss of follow-up was the missing access to immobile patients. Patients had to get access to neurologists' practices. If they could not turn up at the consultations as they were bed-bound at home or in a nursing home, they could not be included and/or further followed in the study. We are aware, that exclusion criteria like dementia or severe depression are a serious limitation for the inclusion of late stage PD patients. However, this ambitious trial addressed successfully with a highly elaborated integrated care program the various needs of PD patients. We are convinced that not the exclusion criteria were the most limiting factor but immobilization of late stage PD patients played a much more important role.

This cohort showed an even lower mortality than the general German population. Patients in the CG had an even lower mortality rate compared to the IG, albeit a lacking individualized therapy. These results are somewhat contradictory and speak for a substantial proportion of patients, who were not been adequately included in this study as we know that mortality normally increases in PD. Another option for the low mortality rates is an overall improved treatment within this study which lead to a better monitoring process in both treatment arms. Patients in both groups were closely monitored in terms of motor functioning, detection of cognitive decline, depression or further non-motor symptoms. It has been shown that a closer monitoring in clinical trials improves patients outcomes (15). The mean disease duration of patients in this study was around 6 years which resembles the lower range of the mean disease duration at death of PD patients (9). This argues for an overall representative group of PD patients in the late stage of the disease, albeit motor symptoms, daily dosage of levodopa or Hoehn and Yahr stage are moderately expressed.

The time of integrating palliative care is critical, especially as in PD/APS many obstacles and preconceptions have to be overcome. The concept of early integration as described by Shin and Temel for oncology patients (16) targets to routinely assess for pain and other symptoms and regularly inquire about a patient's understanding of his disease and his goals of care. This can provide an extra layer of support for patients and their families by helping with more challenging symptom management, psychosocial support, complex decision-making, advance care planning, and transitions in care (16). This concept can easily be adapted to PD patients in order to integrate specialist palliative care at a disease stage at which patients themselves can still decide on their affairs e.g., with respect to advanced care planning like tube feeding, emergency management, future care in a nursing home vs. staying at home etc.

Specialist palliative care is typically accessible for patients with cancer, albeit a variety of measures to improve access to palliative care for people suffering from incurable non-cancer conditions have been implemented more recently. At least shown for Western Australia, in the last 10 years the proportion of patients with non-cancer conditions getting access to specialist palliative care was increasing about 6%. For PD patients this increase was even bigger with 7.5% (17).

There have been some uncertainties, how patients with non-cancer progressive neurological long-term conditions get access to specialist palliative care. Van Vliet et al. reviewed this issue for the UK and found heterogeneity in service provision and integration between neurology and specialist palliative care services, which varied not only between sites but also between diseases (18). Especially PD patients, less APS, did not frequently benefit from specialist palliative care. This asks for integrated care models, e.g. specialist palliative care could be used as an “add-on” approach to the existing integrated care model of the Parkinson's network if needed. Palliative care would then be provided in addition to neurology care, without taking over.

Overall, not only in the late phase, PD patients show an increased utilization of emergency departments. Gerlach et al. reported that 16–45% of PD patients visit the emergency department at least once per year. Additionally, patients were 1.5 fold more likely to be hospitalized and stayed 2–14 days longer than controls (19). Beside the higher rates of hospitalization, symptom burden increases with progressing disease. This leads to a changing role of spouses toward a full-time caregiver. Spouses and family members who form together with the patients the “unit of care,” frequently report to feel isolated and discouraged, without guidance and coordination from healthcare providers and lacking information (20, 21). Finally, they are overstrained after years of supporting and caring for/about the patients. Due to this, a substantial proportion of PD patients dies in hospitals rather than at home or in hospices (12)–even if this is not the preferred place to die for PD patients (22). However, this depends from the symptom burden of patients.

All these findings support the urgent need for advanced care planning (ACP), one important aspect of palliative care. Most of PD patients have not expressed their decisions for proceedings at the end of life. This can include insertion of percutaneous endoscopic gastrostomy (PEG) tube for nutrition as well as the preferred place of death. Overall, reduced (or non-existent) APC in PD patients may lead to an underrepresentation of PD patients in a model of care as presented here. However, APC was not surveyed in this study.

These findings ask for an intensive debate about ACP in PD, as currently it seems not to be adequately addressed during the course of PD. According to Walker, an ACP discussion might “include the individual's concerns, their important values or personal goals for care, their understanding about their illness and prognosis and their preferences for types of care or treatment that may be beneficial in the future and the availability of these” (23). Especially as written ACP are associated with less use of life sustaining treatment, greater use of hospice and less likelihood of hospitalization during end of life phase (24). Furthermore, it was shown, that at least half of PD patients wish to discuss APC early in the course of the disease (25). These findings encourage the implementation of thorough ACP within integrated care structures already at early disease stages.

All these different aspects ask for a further development of the integrated care model, which includes the following principles:

- Integration of specialist palliative care knowledge at a very early point in the course of the disease with respect on the acceptance of the diagnosis (e.g., once a year from the time of the diagnosis),
- Implementation of a *clinical liaison/case manager* (e.g., a PD nurse) as a patient advocate, who takes care of the patient during the course of the disease, especially in critical phases of the disease (e.g., high symptom burden, late stage, etc.)
- Integration of nursing homes, as PD patients in nursing homes are underrepresented in neurological care
- Integration of general practitioners/family doctors, as they have a closer contact to patients' families and know about changing situations of care,
- Dovetailing of neurological and specialist palliative care units and outpatient services in order to use knowledge and the best principles of both disciplines.

## Author contributions

CE: conception, organization, and execution of the research project, data assessment and data analysis, conception and execution of the statistical analysis, writing and critical review of the manuscript drafts. RD: execution of the research project, data management. JS: execution of the research project, data management, statistical analysis. GF: critical review of the manuscript drafts. LT, RV, and HG: data assessment, critical review of the manuscript drafts. SL: conception of the research project, data assessment, critical review of the manuscript drafts.

### Conflict of interest statement

The authors declare that the research was conducted in the absence of any commercial or financial relationships that could be construed as a potential conflict of interest.
